# Neural Plasticity on Body Representations: Advancing Translational Rehabilitation

**DOI:** 10.1155/2016/9737569

**Published:** 2016-09-29

**Authors:** Naoyuki Takeuchi, Shin-Ichi Izumi, Jun Ota, Jun Ueda

**Affiliations:** ^1^Department of Physical Medicine and Rehabilitation, Tohoku University Graduate School of Medicine, 2-1 Seiryo-cho, Aoba-ku, Sendai 980-8575, Japan; ^2^Research into Artifacts, Center for Engineering (RACE), The University of Tokyo, 5-1-5 Kashiwa, Chiba 277-8568, Japan; ^3^George W. Woodruff School of Mechanical Engineering, Georgia Institute of Technology, 771 Ferst Drive, Atlanta, GA 30332-0405, USA

Various physical/mental disorders lead to changes in body representation in the brain that could significantly impact the daily life and function. Advances in noninvasive brain imaging technologies have increased our understanding of neural plasticity that induces functional and structural changes in the central nervous system. Although some neural plasticity aids in the acquisition of new skills and compensates for a loss of function in the body, it has been reported that injury and excessive training drive neural plasticity to maladaptive directions. This neural plasticity is called “maladaptive plasticity” that inhibits complete recovery after injury [1]. This phenomenon is well investigated in body representations after amputation. Maladaptive plasticity after amputation is associated with increased local reorganization within and/or beyond the deafferented sensorimotor cortex [2]. One remote effect of this phenomenon is the reduction of hemispheric asymmetry after amputation, which may reflect the interhemispheric imbalance induced by such reorganization of the deafferented sensorimotor cortex and/or use-dependent changes in the overused intact limb representation.

Investigating neural plasticity after amputation will help facilitate appropriate selection of brain-targeted interventions and reveal cortical reorganization. As a brain-targeted intervention, noninvasive brain stimulation (NIBS) technique alters human cortex excitability, which might lead to adaptive plasticity after amputation. Previous studies revealed that using excitatory NIBS over the deprived sensorimotor cortex improved the phantom pain that was thought to result from maladaptive plasticity [3]. The underlying mechanism for this improvement involves the antalgic effects of the motor cortex stimulation that follow the restoration of the defective intracortical inhibitory processes that appear to be impaired following amputation. Moreover, it is speculated that the analgesic action of the exitatory NIBS to the deafferented motor cortex may be based on the reinforcement of the phantom limb motor representation. On the other hand, the use of inhibitory NIBS over the contralateral deafferented sensorimotor cortex might be effective in decreasing maladaptive plasticity after amputation since the cortical excitability of this cortex might be high due to the excessive nonamputated limb use.

Aside from direct brain stimulation, brain activation feedback might also be effective in correcting maladaptive plasticity and in inducing adaptive plasticity after amputation. Brain-computer interface (BCI) systems record, decode, and translate brain signals for control of a prosthesis without the use of peripheral physiologic activities, bypassing the impaired neuromuscular system. It is postulated that the reestablishment of the disrupted sensorimotor loop by integrating movement intention and passive limb movement assisted by a robot will strengthen associative connections, following the principles of Hebbian plasticity [4].

As described above, various rehabilitation techniques to induce appropriate plasticity on body representation are primarily influenced by learning approaches based on experimental and/or clinical studies. However, these interventions show responses with large interindividual variations. The key to solving this problem is to elucidate the mechanisms by which the human brain adapts to changes in body representation. Body representation consists of body schema, sense of ownership, and sense of agency based on frontoparietal networks. N. Mizuguchi et al. had revealed the neural substrates underlying performance fluctuations using well-trained skillful motor task. They found that fluctuations of the brain activities in the nonmotor frontoparietocerebellar network might underlie the trial-by-trial performance variability even in a well-trained motor skill. Moreover, they showed the possibility that this performance might be affected by the neuromodulation using NIBS.

N. Arizono et al. had presented that functional connectivity is an appropriate biomarker for neurorehabilitation by the sense of ownership in using rubber hand illusion task. They also revealed that there are reliable activity and connectivity from the frontal to the motor related areas during the rubber hand illusion. Thus, these results indicate that the sense of ownership induced by the rubber hand illusion might be related to the neural communication underlying human motor control. In addition, it can be used as a biomarker for the diagnosis of mental disorders or as neurorehabilitation for paralyzed patients.

Information about systematic dysfunction between the nervous system and the body is invaluable for accurate, valid assessment of disabling neurological deficits and for the development of treatment methods. M. Pazzaglia and M. Zantedeschi had reported that body awareness is synthesized from multimodal integration and temporal constancy of multiple body representations. Further, they reviewed the following points: (a) the precision in the body metric may be based on the sight and positioning sense of a particular body segment, (b) body awareness reflects an innate organizational experience of unity and continuity in the brain, and (c) body awareness is based on efferent/afferent neural signals. They also discussed the abnormalities and therapeutic strategies for correcting bodily distortions in spinal cord injury. In addition, they found that the neural maladjustment of body representation and/or abnormalities in somatognosia can occur independently from the dysfunction. This indicates that creating and maintaining body representation are essential for appropriate function.

The purpose of this special issue was to describe the neural mechanisms of body representation in the brain and the mechanisms underlying the long-term changes in representation, which can be useful in the application of rehabilitation interventions. Since these approaches can be targeted to specific impairments and needs, these can be considered as “personalized interventions.” The studies concerning different etiologies of neurological deficits and those focused on the behavior of healthy subjects are useful in this topic as long as they serve the primary goal of advancing a theoretical and translational framework for neurorehabilitative practice. In the future, combining brain science and rehabilitation medicine by using systems engineering is anticipated to yield translational outcomes.
